# Avoiding the distant elephant: a model to approach the research component of specialization

**DOI:** 10.1186/s12909-016-0661-x

**Published:** 2016-05-14

**Authors:** Colleen Aldous, Damian Clarke, Jacky van Wyk, Chris Rout

**Affiliations:** School of Clinical Medicine, College of Health Sciences, University of KwaZulu-Natal, Durban, South Africa; Department of Surgery, School of Clinical Medicine, College of Health Sciences, University of KwaZulu-Natal, Durban, South Africa; Department of Clinical and Professional Education, School of Clinical Medicine, College of Health Sciences, University of KwaZulu-Natal, Durban, South Africa; Department of Anaesthetics, School of Clinical Medicine, College of Health Sciences, University of KwaZulu-Natal, Durban, South Africa

**Keywords:** Research, Mentorship, Strategy, Model, Research specialization, Supervision

## Introduction

In January 2010 the Health Professions Council of South Africa’s (HPCSA) Medical Subcommittee for Postgraduate Education and Training published new requirements for specialist registration [1]. The requirements included the completion of a research project, resulting in publication or a successfully examined dissertation. Since 2011 therefore, all specialist trainees registered with the various Colleges of Medicine of South Africa (CMSA, the examining body), have had to fulfill the above research requirement in addition to clinical specialist training in an approved centre, production of a satisfactory portfolio of clinical casework, and successful completion of the CMSA exams.

Before 2011 there were two routes to specialist registration in South Africa; some Medical Schools used a Masters degree (MMed, including the requirement for a research project) awarded by the parent University after successful completion of an internal examination, others used the prescribed syllabus of the relevant College of Medicine (which included research methodology, statistics and clinical trial design but without the requirement for a research project) followed by successful completion of the College Fellowship examinations (common to all centres). All applicants for specialist registration also had to receive a certificate of satisfactory completion of training signed by both the head of the relevant specialty discipline and the Dean of the School of Medicine.

Effectively the new regulations mean that the successful postgraduate student now completes training with the dual qualifications of MMed and CMSA Fellowship before HPCSA specialist registration.

Because the MMed is a University degree, supervision of the research project falls under the regulations of the South African Committee for Higher Education (CHE), which stipulate [2] that:

“…in addition to their being acceptable to the research community, must include the following:

- The supervisor has a qualification in a relevant field of study higher than, or at least at the same level as, the exit level of the postgraduate programme he/she is supervising.
- The supervisor has an appropriate research track record, as well as experience, expertise and peer recognition in the field of study.
- In the case of inexperienced or new supervisors, there is ongoing staff development and support, and joint supervision is explored as an option.”

These regulations have created a problem in those centres that previously pursued the CMSA Fellowship route in that the CHE does not regard the CMSA as an educational body and thus does not recognize the Fellowship examination as “a qualification”. Without the history of production or employment of staff with a Master’s degree or higher, there is a paucity of suitably qualified supervisors. Those with a Master’s degree may not have the research track record or supervision requirements stipulated in the other bullets.

The research study is allocated a minimum of 60 credits in terms of the Standards Generating Body of the Medical and Dental Board of the HPCSA. The South African Qualifications Authority (SAQA) equates one credit with 10 notional hours of learning [3] that are supposed to approximate the time the student requires to achieve learning outcomes, assuming a 45-week full-time academic year. In the case of the research component of both HPCSA and CHE regulations, this amounts to a minimum of 600 h’ work (equivalent to 75 8-h working days). It has been argued [4] that the required time for a Master’s degree is closer to 4000 h. In either case the hours have to be fitted into an already busy schedule of clinical practice, knowledge acquisition and preparation for two (sometimes three) examinations (Primary and Final with an Intermediate examination in surgical disciplines) over 4 years.

Wingfield has also argued that to supervise a research project takes about 10 % of the time that the student has to commit to complete the degree [5]. Teaching a student how to ask a relevant research question, do a literature review, prepare a research protocol, get them through their first experience of research and produce either an examinable dissertation or publishable research article would be difficult under this time constraint. To fit this extra task into the 4-year specialist training period poses time challenges to both students and supervisors. Before the research component of specialization became compulsory, the MMed reportedly started out as the qualification with the lowest completion rate in 2011 because of the time issue [6].

Additional potential problems for the new student are the lack of a full appreciation of possible areas of interest in which to pursue research within the specialty and the relatively long time-frame in which all components for registration have to be completed (the distant elephant in the metaphor).

The above time estimates were based upon the traditional Apprentice Master Model (AMM) i.e. where a supervisor would guide the student through the process [7]. At the time of the implementation of the MMED qualification, postgraduate students at most institutions of higher learning were supervised in the AAM. The cohort model has since emerged as a viable alternative to traditional supervision and promises to use supervisors’ strengths and time in a more creative manner.

In the absence of a structured collaborative cohort model [8] in the School of Clinical Medicine (SCM), University of KwaZulu-Natal (UKZN), two of the authors (DC and CM), as disciplinary supervisor and research mentor to five MMed students registered in 2011 (all of whom had graduated with relatively limited research experience from their respective undergraduate programmes) developed a modular approach to progression within the research component to prevent the “distant elephant” syndrome, ensuring timeous completion of the degree within the newly specified regulations.

This paper describes a modular system to assist five MMed students from the first cohort for whom the research component was compulsory for registration as a specialist. They were registered with UKZN in the Department of Surgery of the Pietermaritzburg Metropolitan Complex of hospitals from 2011 to completion of training in 2014. An ethics waiver from the universities’ Biomedical Research Ethics Committee was obtained in order for this article to be written. As individual students are not mentioned, no consent was required.

### The presentation of material

The module was offered in addition to a 2-day SCM research methodology course open to all novice researchers at the time. The supervisor, a specialist surgeon took responsibility for surgical aspects of the research. The research mentor took responsibility for scientific process, including the design of protocol templates and an appropriate textual source [9]. Both took responsibility for quality control of research outputs.

The modular approach broke the research process into its component elements, which would fit comfortably into the allocated 4 year training period. The elements reflected requisite milestones in the research process, facilitated by accompanying task-specific templates aimed at the required output. These were timetabled into a 4-year time frame divided into 16 quarters; the supervisors provided support and guided the pace for students throughout the project timeline in general, and specifically between each milestone to ensure successful completion.

Two constraints placed upon the design of the programme had to be accommodated when planning for the students to achieve all the required training outcomes as for as a specialist; these were:

- The same 4 year training period had been allocated as pertained before the new regulations,
- The research project had to be completed (acceptance for publication) within the stipulated period.

To ensure that registrars could complete a research project competently within this timeframe, the timeline was developed to dovetail the research into the 4 years with the other learning, training, clinical, and examination commitments. Instructive templates were designed for and used with each phase of the process e.g. elements of protocol development were taught while students wrote their protocols, scientific writing whilst preparing for publication and oral conference presentation.

#### The timeline

**Fig. 1:**
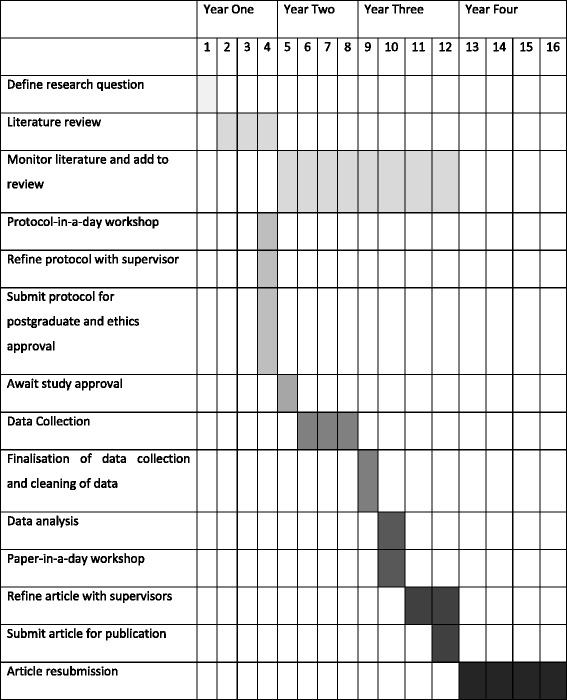
Gantt chart for MMed research project process through 16 year quarters

The entire research project had to proceed alongside preparation for the Primary examinations that are taken in Year 1 and Final examinations taken in the fourth year. A Gantt chart (Fig. 1) shows the timeline designed to accommodate the research process. A research area first had to be chosen to facilitate an appropriate literature search and formulate the research question. Much of the first year was allocated to literature collection to minimize the research project workload as students prepared for the Primary examination. An initial period of 3 months post registration was therefore allocated to allow students to become sufficiently orientated within the clinical environment to identify possible areas of research interest.

Data collection, analysis and the first draft of the paper were scheduled to be completed by the end of the third year, leaving the fourth year free for preparation for the Final examinations. Acceptance for peer-reviewed publication of the paper in a peer-reviewed format was taken as completion of the research component for this group of students.

#### The templates

Two templates were developed to facilitate a streamlined learning process enabling students to complete their research as independently as possible within the constraints of the limited time available for sufficiently detailed teaching of research methodology.

##### The protocol template

The protocol template contained all the elements of a general protocol for a quantitative study. Blocks of instruction were combined with written examples for guidance and the student then completed a section using material relevant to their own studies, deleting the instructional blocks as they progressed. The research mentor used a workshop format i.e. a “Protocol-in-a-day”, to guide students through the protocol development process. The relevance of each section was explained and the scope of possible materials and the rationale for their use were developed for different study contexts. The Protocol-in-a-day workshop was offered after the initial literature review had been completed so that it could be pasted into the protocol during the workshop.

##### The article template

The article template consisted of a booklet of worksheets covering the writing of the introduction, methods, results and discussion of a study followed by the writing of the abstract. Notes were included on the requirements for each section and guided the writing process. The methods section could be lifted from the well-written protocol and the results section was a process of insertion of the observed data and analysis. All the other sections were structured around a series of questions or instructions that had to be completed in sequence. At a “Paper-in-a-day” workshop, the research mentor facilitated the students’ writing of all sections in a single day in order to produce a first draft of their article. After the workshop, the students added finishing touches such as the list of references, refined the article and then worked on final presentation for the journal with the supervisor.

### Outcomes and discussion

By September of 2014, two of the students’ research reports were in print as journal articles, one was accepted for publication, and two had been submitted for review at least 5 months prior and were still in the review process. At the end of the 4 year training period, three of the papers were in print and two had been accepted for publication, and all five cohort members had passed all their CMSA examinations, thus achieving HPCSA requirements for specialist registration.

The inclusion of a research project as an obligatory requirement for specialist registration, including those students pursuing the CMSA examination pathway, has met with resistance in some quarters. Amongst the trainees themselves was the belief or hope that the HPCSA would change their minds and drop the research component requirement. This resulted in procrastination and inadequate preparation for some, leading to delayed progress. This led to the belief that many registrars who started their training in 2011 would not have completed their research in time for registration having passed their final exams. While a final analysis on the impact of the changed regulations on specialist registration has yet to be published, the authors are aware of a few instances where this has been the case.

The imposition of extra workload upon students and supervisors without acknowledgement of the time required to achieve the desired outcome against a background of inadequate numbers of supervisors meeting CHE requirements (in centres lacking a history of a research-based MMed programme) has led to justifiable critical resistance [10], that has yet to be addressed. However, we have shown that expedient management of the research process and the use of appropriate templates in co-supervision management of a student cohort can achieve an externally motivated goal in the time available. Application of this model has the added advantages of decreasing the isolation a student might otherwise feel and improves the quality of scientific writing, contributing to successful completion of the research project [11].

The close collaboration between the research mentor, managing the research process, and the clinical supervisor, managing the surgical aspects of the projects ensured that students were guided and supported through the full scope of the endeavour. The process included formal planning of time management, structured formats to assist first drafts of both the protocol and paper, and finally assistance with submission to and communication with the targeted journal. This started from topic selection and inception of the project through to project implementation and analysis. While we had the advantage of a small group of students in a single discipline, this method could easily be extended to larger cohorts of students across disciplines with a clinical supervisor for each discipline. Although timetabling logistics might prove difficult (even in this cohort there were times when not everyone could attend), within a 4 year window there is always a way to make a plan.

The curriculum structure offered flexibility, with milestones being slotted into year quarters allowing planning for group sessions whenever possible. The challenge presented by clinical rotations and irregular work shifts was overcome by occasional repetition of some of the sessions. Although this increases the time cost to the supervisors, this is nothing in comparison to the costs of the students not completing the work in the time allotted. Again, the process was facilitated by the partnership between the clinical and research supervisors.

The correct combination of talents is important in the supervisory team. The research mentor should have a broad knowledge and experience of different research methodologies and study designs, and should have the ability to teach necessary skills, relevant to the projects, without an overly obsessive approach to process. It is important to remember that the purpose of the exercise is to fulfil the research component of specialist registration requirements; few, if any, of the students will progress to become researchers but the exercise should be a positive experience through which they feel they might.

The clinical supervisor should be familiar with the broad field of study and its literature to the extent that guidance can be provided in the choice of narrower individual research questions and also familiar with local clinical load in the context of patient numbers available for study, allowing rapid data accrual. We were fortunate in that the clinical supervisor for this cohort had been part of the development of a clinical database for trauma surgery for the Pietermaritzburg Metropolitan Complex of Hospitals [12] and therefore had access to data that could be used to answer several epidemiological questions suitable as research topics. The projects met the criteria of being clinically focused and addressing common conditions in the trainees’ discipline, both of which can be challenging, and also addressed the paucity of clinical reports of frequently encountered surgical conditions seen in developing world practice of relevance in the developed world.

Regular contact session between the students and the supervisors enabled a close monitoring of the students’ progress throughout. This was facilitated by having the surgeon as supervisor within the same department as the students and enabled frequent informal communication around the project. The scientific advisor made bi-weekly visits to the campus and maintained e-mail availability at all times. Personal involvement with effective use of the above supervisory and mentoring method and the expedient use of templates contributed to the timely completion of the projects.

This first attempt at getting students to complete their research projects within their 4 years has allowed the supervisors to identify some possible improvements. The authors were part of the development of the database for trauma in the Metropolitan Hospital Complex [12], which had been granted class approval by the University Biomedical Research Ethics Committee (BREC). Following notification of BREC for individual projects, students could access the database for their MMeds. This obviated the process of obtaining full ethical approval for each of the studies, which now required only postgraduate approval. This reduced delay in the fifth quarter when the students awaited approval before commencement of the study. It also reduced the time required for data collection within the allotted 9 months. Access to a database which has class approval can therefore reduce the time it takes the student to submit a paper from 3 years to just over 2 years.

We have looked at only one cohort of students, the first to complete training under the new HPCSA regulations. While this cohort of surgical trainees represents a success story, we recognize that other disciplines may not be able to implement this curriculum in its current form. However, the research mentor also assisted an internal medicine and an anaesthesia trainee using the same process and materials, and they also achieved publication of their studies and specialist registration within the prescribed 4 year period. So the methods used were not discipline specific, but some modification of the Gantt chart might be necessary to accommodate the different placement of milestones between disciplines.

## Conclusion

In conclusion, the success of the five specialist trainees in achieving the MMed at our institution within the allotted 4 years has given us confidence in the approach we have taken. We encourage development of databases to streamline ethics approval, ease the burden of data collection, and direct relevant research-based improvements to the care of our patients. It remains to be seen whether collaborative cohort supervision can be successfully applied across disciplines at the Masters level.

## Abbreviations

AMM: apprentice master model
CHE: Committee for Higher Education
CMSA: Colleges of Medicine of South Africa
HPCSA: Health Professions Council of South Africa
MMed: master of medicine
SAQA: South African Qualifications Authority
UKZN: University of KwaZulu-Natal
